# Dynamics of human serotonin synthesis differentially link to reward anticipation and feedback

**DOI:** 10.1038/s41380-024-02696-1

**Published:** 2024-08-23

**Authors:** Andreas Hahn, Murray B. Reed, Matej Murgaš, Chrysoula Vraka, Sebastian Klug, Clemens Schmidt, Godber M. Godbersen, Benjamin Eggerstorfer, David Gomola, Leo R. Silberbauer, Lukas Nics, Cécile Philippe, Marcus Hacker, Rupert Lanzenberger

**Affiliations:** 1 Department of Psychiatry and Psychotherapy, Medical University of Vienna, Vienna, Austria; 2 Comprehensive Center for Clinical Neurosciences and Mental Health (C3NMH), Medical University of Vienna, Vienna, Austria; 3 Department of Biomedical Imaging and Image-guided Therapy, Division of Nuclear Medicine, Medical University of Vienna, Vienna, Austria

**Keywords:** Neuroscience, Physiology

## Abstract

Serotonin (5-HT) plays an essential role in reward processing, however, the possibilities to investigate 5-HT action in humans during emotional stimulation are particularly limited. Here we demonstrate the feasibility of assessing reward-specific dynamics in 5-HT synthesis using functional PET (fPET), combining its molecular specificity with the high temporal resolution of blood oxygen level dependent (BOLD) fMRI. Sixteen healthy volunteers underwent simultaneous fPET/fMRI with the radioligand [^11^C]AMT, a substrate for tryptophan hydroxylase. During the scan, participants completed the monetary incentive delay task and arterial blood samples were acquired for quantifying 5-HT synthesis rates. BOLD fMRI was recorded as a proxy of neuronal activation, allowing differentiation of reward anticipation and feedback. Monetary gain and loss resulted in substantial increases in 5-HT synthesis in the ventral striatum (VStr, +21% from baseline) and the anterior insula (+41%). In the VStr, task-specific 5-HT synthesis was further correlated with BOLD signal changes during reward feedback (ρ = −0.65), but not anticipation. Conversely, 5-HT synthesis in the anterior insula correlated with BOLD reward anticipation (ρ = −0.61), but not feedback. In sum, we provide a robust tool to identify task-induced changes in 5-HT action in humans, linking the dynamics of 5-HT synthesis to distinct phases of reward processing in a regionally specific manner. Given the relevance of altered reward processing in psychiatric disorders such as addiction, depression and schizophrenia, our approach offers a tailored assessment of impaired 5-HT signaling during cognitive and emotional processing.

## Introduction

The adequate processing of reward and punishment constitutes crucial aspects of brain function and mental well-being. Accordingly, altered reward processing and anhedonia represent core symptoms in various brain disorders, particularly in addiction [1], depression [2, 3] and schizophrenia, underlining its clinical relevance [4]. The reward circuitry involves a complex network of interactions, with dopaminergic innervation of the nucleus accumbens/ventral striatum (VStr) by the ventral tegmental area at its core [5–7]. The VStr in turn spans a complex network of connections with various other sub-/cortical regions [5], responsible for encoding different components of reward processing, such as anticipation (wanting) and hedonic experience (feedback, (dis)liking) [6].

In addition to dopamine, the serotonin system plays a crucial yet often overlooked role in reward processing [8]. However, investigating serotonergic neurotransmission in the human brain during cognitive and emotional stimulation poses significant challenges [9, 10]. While positron emission tomography (PET) is an established tool for imaging dopamine release with the competition model [11], current radioligands for the serotonin system require potent pharmacological manipulations to induce binding differences [12–14]. In contrast, identifying subtle changes induced by task performance is considerably difficult, with only one study reporting changes in radioligand binding during visual stimulation [15]. However, effects were limited to a few percent and not specific to brain areas involved in task processing.

**Fig. 1:**
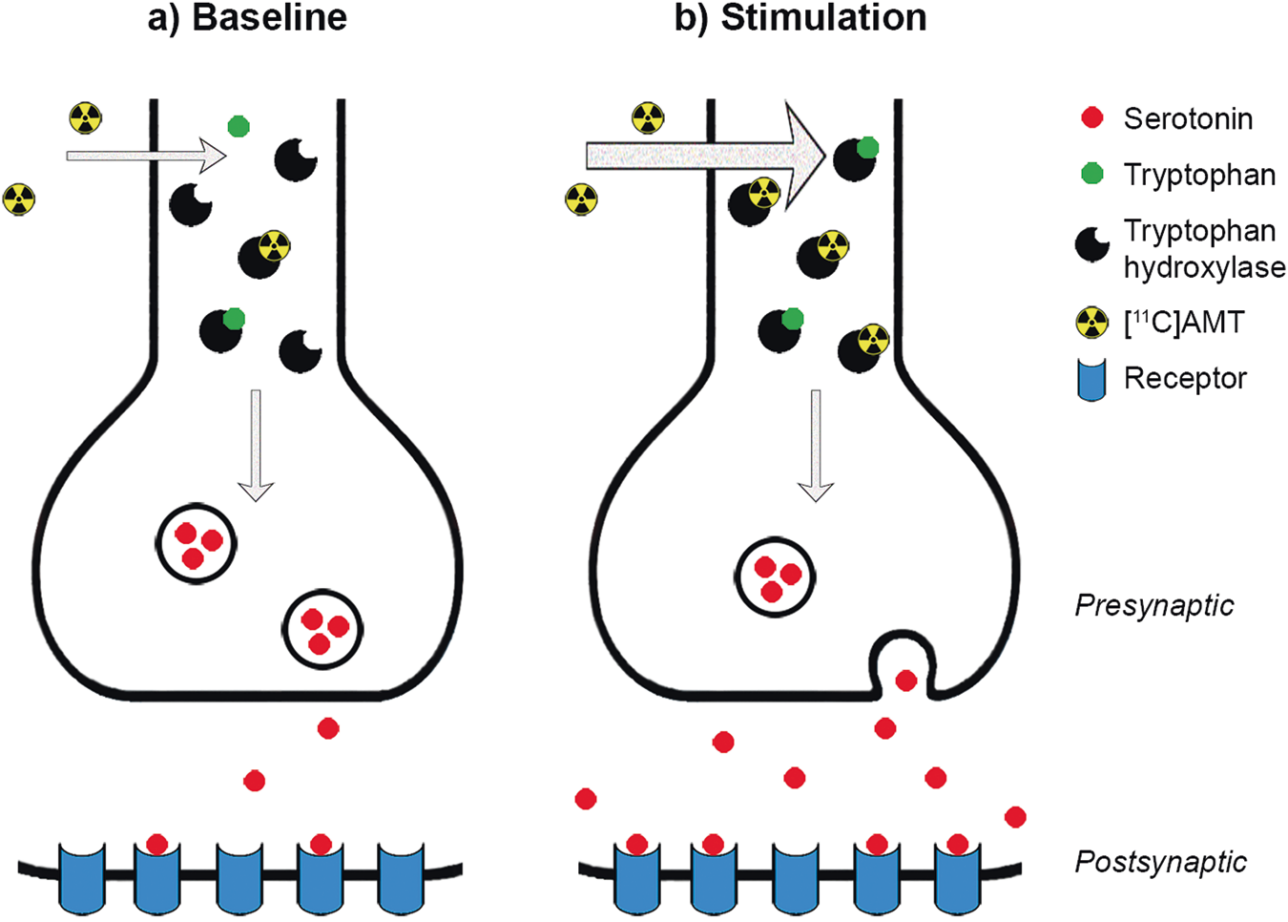
Schematic description of the synthesis model in the context of the serotonin system. **a** Tryptophan is converted to 5-hydroxytryptophan (5-HTP) by the enzyme tryptophan hydroxylase, followed by conversion to serotonin (5-HT) via amino acid decarboxylase. As the radioligand [^11^C]AMT is a substrate for the first step of the synthesis, its use represents an established approach to image 5-HT synthesis rates at resting state. **b** Neuronal stimulation elicits serotonin release, which also increases the activity of 5-HT synthesis enzymes to refill synaptic vesicles with newly synthesized neurotransmitter. This in turn also leads to an increased radioligand uptake. Figure adapted from [16] under CC-BY license.

Functional PET (fPET) represents a promising technique to investigate changes in neurotransmitter action upon stimulation. The synthesis model [16] proposes that neuronal firing also affects neurotransmitter synthesis in order to replenish the synaptic vesicles after stimulation-induced neurotransmitter release (Fig. 1). Our group successfully demonstrated corresponding changes in radioligand binding for dopamine synthesis [16]. Similar to dopamine, also serotonin cannot cross the blood brain barrier (BBB), therefore it is synthesized within the brain. The main synthesis pathway is given by the conversion of tryptophan to 5-HTP via the enzyme tryptophan hydroxylase, and subsequently to 5-HT via amino acid decarboxylase. Crucially, fast-acting regulatory mechanisms control the activity of these enzymes. That is, tryptophan hydroxylase activity increases during neuronal stimulation [17, 18] and fenfluramine-induced serotonin release increases serotonin synthesis [19]. The radioligand alpha-[^11^C]methyl-L-tryptophan ([^11^C]AMT) represents a validated approach to image 5-HT synthesis rates at baseline in the human brain since it is a specific substrate for tryptophan hydroxylase and is therefore incorporated into the synthesis chain [20–22].

Here, we aimed to extend the synthesis model within the fPET framework to investigate serotonin-signaling dynamics during reward processing. We hypothesize that task performance induces increases in [^11^C]AMT binding, thereby providing an imaging marker of stimulation-specific serotonergic activity in the living human brain. Given the complex interaction of numerous brain regions [5, 6] and the specific involvement of the 5-HT system in reward [8], we expect changes in 5-HT synthesis beyond the VStr. Finally, we combine the molecular specificity of PET with the high temporal resolution of BOLD fMRI to link 5-HT signaling with distinct phases of reward processing.

## Methods

### Participants

For this study, we initially recruited 30 healthy volunteers. However, data of seven participants was not available due to failure of radioligand synthesis and four due to problems with arterial cannulation. Additionally, two volunteers dropped out for personal reasons before the PET/MR scan. One subject was excluded from the results because changes in 5-HT synthesis were three standard deviations apart from the group average. Thus, the final sample comprised 16 healthy participants (mean age ± standard deviation = 23.4 ± 3.7 years, 8 females). As this is the first study investigating task-specific effects in 5-HT synthesis with PET, sample size was based on our previous work [16]. At the screening visit all participants underwent a routine medical examination by an experienced psychiatrist. This included assessment of general health through medical history, physical examination, blood tests, electrocardiography, neurological testing and the Structured Clinical Interview for DSM-5. Exclusion criteria were current and previous somatic and neurological disorders as well as substance abuse and neuropsychopharmacological treatment. Furthermore, contraindications for PET (study-related radiation exposure within the last 10 years) and MRI scanning (metal implants, claustrophobia), pregnancy and breast-feeding were reasons for exclusion. Female participants underwent a urine pregnancy test at the screening visit and before the PET/MR scan. All subjects provided written informed consent after the detailed explanation of the study protocol, and were insured and reimbursed. The study was approved by the ethics committee of the Medical University of Vienna (ethics nr: 2259/2017) and all procedures were carried out according to the Declaration of Helsinki.

### Cognitive task

Reward processing was induced by the monetary incentive delay (MID) task [16] designed to maximize gain and minimize loss. In short, participants were presented with a cue displaying a certain monetary amount (±0.5, 1 or 3 €) for a few seconds, followed by the target stimulus (!). Participants were instructed to press a button as quickly as possible. Subsequently, feedback was provided, indicating whether gain or loss occurred and the current accumulated amount of money. The paradigm consisted of four task blocks (2× gain, 2× loss), each lasting for 5 min and comprising 27 trials.

The critical part of the paradigm lies within the manipulation of the reaction time, as described previously [16]. Essentially, the individual reaction time was determined as an average of eight training trials, conducted immediately before the PET/MR examination. At the beginning and in the middle of each task block, the reaction time limit was decreased (or increased) to change the probability of the current block towards monetary loss (or gain). Additionally, the reaction time limit was adaptively modified based on performance, i.e., increased (or decreased) if participants were too slow (or sufficiently fast). This gradually changes the probability of success towards 0.5, decreasing the participants’ awareness of the manipulation.

### PET/MR data acquisition

Simultaneous PET/MR imaging was carried out with a hybrid mMR scanner system (Siemens Healthineers). First, a T1-weighted structural MR image was recorded (MPRAGE sequence, TE/TR = 4.21/2200 ms, TI = 900 ms, flip angle = 9°, matrix size = 240 × 256, 160 slices, voxel size = 1 × 1 × 1.1 mm, TA = 7:41 min), which was used to obtain a pseudo-CT for attenuation correction and for later spatial normalization of functional images. After that, 50 min of fPET acquisition simultaneously began with the application of the radioligand [^11^C]AMT (10 MBq/kg body weight, 5.4 µSv/MBq [21]) with the infusion pump (Syramed µSP6000) placed in an MR-shield (UniQue, Arcomed). 20% of the activity was injected as a bolus, while the rest was administered as constant infusion until the end of the scan [23]. The 5-min MID task blocks started 10, 20, 30 and 40 min after start of the radioligand application. During each task block, BOLD fMRI was obtained in parallel to fPET (EPI sequence, TE/TR = 30/2000 ms, flip angle = 90°, matrix size = 80 × 80, 34 slices, voxel size = 2.5 × 2.5 × 2.5 mm + 0.825 mm gap, TA per block = 5:30 min). During all the resting periods, a crosshair was presented and subjects were instructed to keep their eyes open and let their thoughts come and go freely/avoid focusing on anything in particular.

### Blood sampling

Manual arterial blood samples were taken from the radial artery every 20 s for the first three minutes and then at 3, 4, 5, 16, 26, 36 and 46 min (i.e., during periods of rest). The activity of whole blood and plasma (after centrifugation) was measured in a gamma counter (Wizard 2, 3”, Perkin Elmer). To obtain the arterial input function, plasma to whole-blood ratios were fitted with a linear function, which was multiplied with the whole-blood data.

### Quantification of serotonin synthesis rates

fPET images were corrected for attenuation with a pseudo-CT [24] and reconstructed to frames of 30 s. Image preprocessing was carried out as described previously [16, 23, 25] in SPM12 using default parameters unless specified otherwise. This included motion correction (quality = best, register to mean) and spatial normalization to MNI stereotactic space via the T1-weighted structural image. Images were smoothed with a sliding window non-local means filter [25] as well as subsequent 5 mm Gaussian smoothing and application of a temporal low-pass filter with (1/150 s cut-off frequency). This strategy increases the signal to noise ratio, while maintaining a high test-retest reliability of fPET data [26].

Identification of task-specific changes in 5-HT synthesis rates was done with the general linear model as established previously [16, 23]. The design matrix included one regressor to account for baseline effects, one for each task condition (gain and loss) and additional regressors to correct for head motion. The baseline regressor was defined as the average time activity curve across all gray matter voxels, excluding those voxels that were declared as activated by the fMRI acquisition (p < 0.001 uncorrected) and those that were identified in a recent meta-analysis [27]. The task regressors were defined as a ramp function with a slope of 1 kBq/frame. The motion regressors were given by the first principal components of the six realignment parameters, with the number of relevant components identified by the elbow criterion [25].

Combining output from the GLM and the arterial input function, the net influx constant K_i_ was then quantified with the Gjedde-Patlak plot separately for baseline and task conditions. This represents a robust index of 5-HT synthesis rates [21, 22]. In addition, we computed percent signal changes of task effects relative to baseline as

1 $$\% {{\rm{SC}}}={{{\rm{Ki}}}}_{{{\rm{task}}}} / {{{\rm{Ki}}}}_{{{\rm{baseline}}}}\,* \,100$$

### BOLD-derived neuronal activation

Task-induced neuronal activation was inferred from BOLD fMRI signal changes as estimated with SPM12 [16]. Images were corrected for slice timing differences (reference = middle slice) and head motion (quality = best, register to mean), followed by spatial normalization to MNI space and smoothing with an 8 mm Gaussian kernel. To separate reward anticipation and feedback, different regressors were included in the GLM analysis. These were one for each cue (gain, loss), one for the target stimulus and one for each of the different outcomes (gain, omitted gain, loss, avoided loss) as well as several nuisance regressors (signals from white matter and cerebrospinal fluid, motion parameters). The contrasts of interest for the group analysis were anticipation of gain and loss, feedback of success (gain + avoided loss) and feedback of failure (omitted gain + loss).

### Statistical analysis

The primary region of interest was the ventral striatum/nucleus accumbens (VStr) due to its central role in reward processing [5]. Thus, 5-HT synthesis rates of the VStr were extracted using the Harvard-Oxford atlas. Task-induced changes in K_i_ were assessed with one sample t-tests against zero separately for monetary gain and loss. Differences between gain and loss conditions were tested with a paired t-test. Considering previously reported sex-specific effects in dopamine synthesis during reward processing [16], we also tested these differences separately for women and men as well as a sex by condition interaction. However, considering the lack of differences between monetary gain and loss (see “Results”), these two conditions were combined for all subsequent fPET and fMRI analyses.

As the radioligand [^11^C]AMT also shows good signal to noise ratio in cortical regions, an exploratory voxel-wise analysis was conducted on 5-HT synthesis changes in SPM12. We aimed to avoid spurious findings potentially attributed to the low sample size. Thus, an initial voxel threshold of p < 0.0001 uncorrected was applied, followed by a cluster threshold of p < 0.05 FWE corrected. Furthermore, post-hoc analysis of significant clusters and interpretations were limited to brain regions involved in reward processing [27].

BOLD signal changes were extracted for the a priori defined (VStr) and exploratory identified brain regions (anterior insula, see results), and significance was assessed with one-sample t-tests against zero. To link changes in 5-HT synthesis with the specific reward processing phases (i.e., anticipation and feedback), Spearman correlations were conducted between fPET and fMRI parameters. To test whether either of these variables were able to explain individual differences in the reaction time of the reward task, changes in 5-HT synthesis and the BOLD signal were entered in a multivariable regression analysis.

Data were inspected to meet a normal distribution and all statistical tests were two-sided. Correction for multiple comparisons (conditions, brain regions, voxels) was done with the Bonferroni–Holm procedure (p_BH_) and FWE cluster correction (p_FWE_, see above) for ROI-based and voxel-wise analyses, respectively.

## Results

### Behavioral data

On average, participants won 6.9 ± 2.1 € and lost −5.6 ± 2.6 € during the corresponding task blocks (all p < 10^-6^), confirming successful task performance. Reaction times were slightly higher for monetary gain (392.7 ± 36.5 ms) than for loss (385.4 ± 36.3 ms, p < 0.05).

#### Serotonin synthesis

**Fig. 2:**
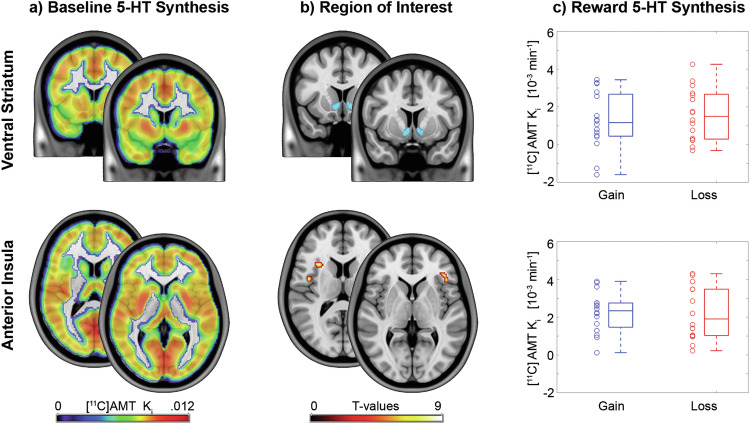
Serotonin (5-HT) synthesis in the ventral striatum (VStr, top row) and anterior insula (bottom row). **a** Baseline serotonin synthesis as obtained with [^11^C]AMT. **b** Regions of interest indicate the anatomical definition of the VStr of the Harvard-Oxford atlas (“nucleus accumbens” marked in blue, top) and the anterior insula as obtained from the voxel wise analysis on task-specific changes in 5-HT synthesis (p < 0.05 FWE corrected cluster level, t-values in red-yellow, bottom). **c** Reward-specific changes extracted from the regions of interest (**b**) of the VStr (top) and anterior insula (bottom) during monetary gain (blue) and loss (red). There was no significant difference between monetary gain or loss (all p > 0.2). Axial and coronal slices (**a**, **b**) are shown at z = 3/13 mm and y = 8/14 mm MNI space, respectively. Boxplots (**c**) indicate median values (center line), upper and lower quartiles (box limits) and 1.5*interquartile range (whiskers).

fPET showed an increase in [^11^C]AMT 5-HT synthesis in the VStr (Fig. 2c) during the two experimental conditions of monetary gain (K_i_ = 0.0013 ± 0.0015 min^-1^, p_BH_ < 0.01; %SC = 21.2 ± 32.6%) and loss (K_i_ = 0.0016 ± 0.0014 min^-1^, p_BH_ < 0.001; %SC = 21.6 ± 19.8%). In contrast to our previous work on dopamine synthesis [16], there was no significant difference between monetary gain and loss neither for the entire group (p = 0.5), nor for men (p = 0.9) or women separately (p = 0.2). There was also no sex by condition interaction (p = 0.5).

In addition to the primary region of interest, a voxel-wise analysis was carried out to identify task-induced changes in 5-HT synthesis beyond the VStr. This revealed a significant bilateral increase in 5-HT synthesis in the anterior insula (p_FWE_ < 0.05, Fig. 2c). For the combined contrast of gain and loss, this was K_i_ = 0.0023 ± 0.0011 min^-1^, p < 10^−6^, corresponding to 40.6 ± 27.2% change from baseline.

#### Neuronal activation

BOLD fMRI was then used to investigate the temporal specifics of neuronal activation. As expected, this resulted in significant BOLD signal changes in the VStr during reward anticipation (p_BH_ < 10^−6^) and feedback (p_BH_ < 10^−4^). For the anterior insula, there was a significant increase in the BOLD signal during anticipation (p_BH_ < 0.001), but not for feedback (p_BH_ = 0.12).

#### Multimodal associations

**Fig. 3:**
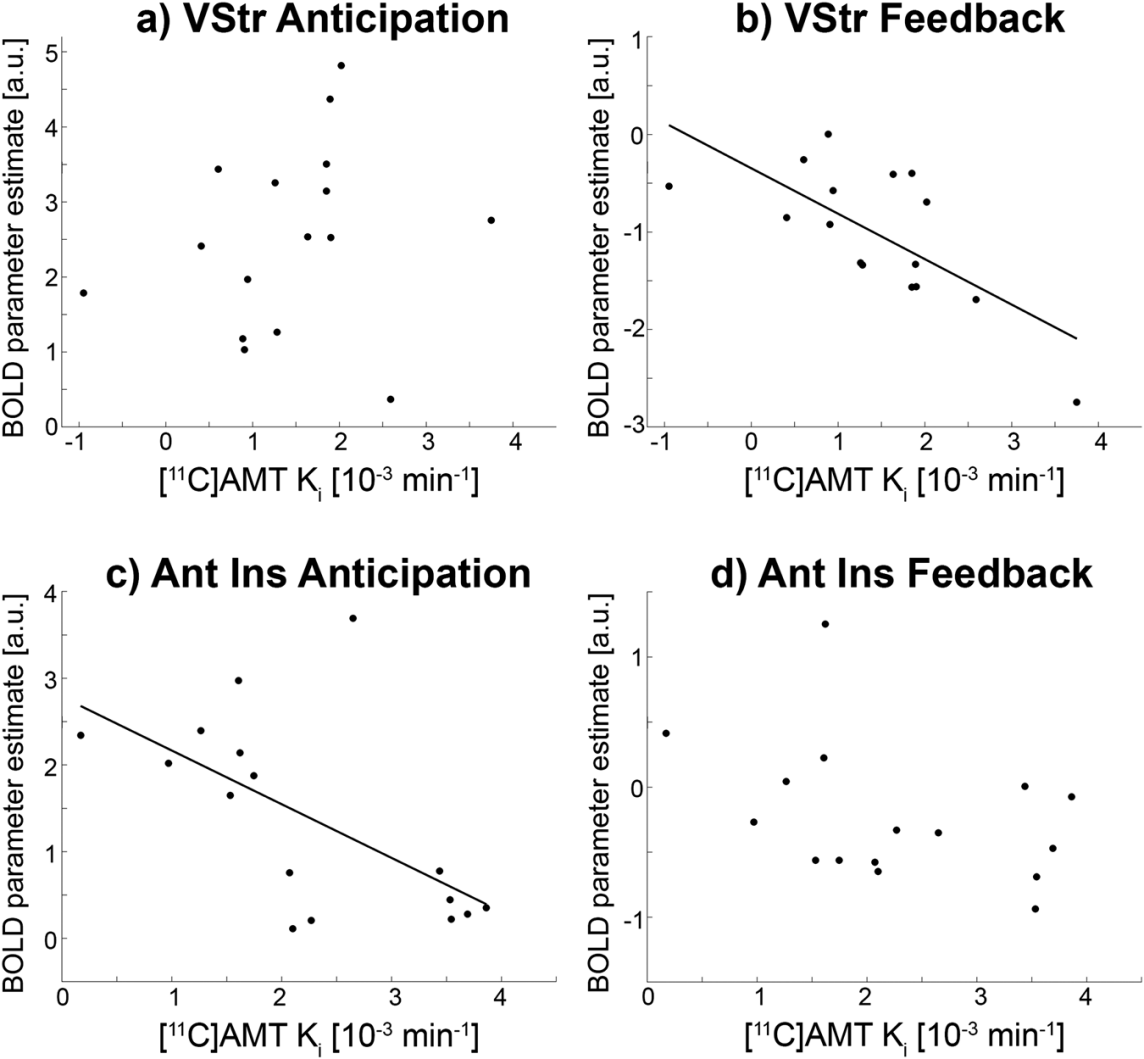
Associations between fPET and fMRI during reward anticipation and feedback. Task-specific [^11^C]AMT serotonin synthesis of the ventral striatum (VStr) was negatively associated with BOLD fMRI signal changes during reward feedback (**b**, ρ = −0.65, p_BH_ < 0.05), but not during anticipation (**a**, ρ = 0.3, p_BH_ = 0.26). On the other hand, serotonin synthesis of the anterior insula (Ant Ins) was negatively related to BOLD signal changes of reward anticipation (**c**, ρ = −0.61, p_BH_ < 0.05), but not reward feedback (**d**, ρ = −0.45, p_BH_ = 0.16). K_i_ represents the net _i_nflux constant of [^11^C]AMT serotonin synthesis rates in units of min^−1^. BOLD parameter represent the result of the general linear model in arbitrary units.

Next, we aimed to link the temporal dynamics of neuronal reward processing to changes in 5-HT synthesis. Direct comparison between fPET and fMRI showed a negative association between VStr 5-HT synthesis and BOLD signal changes for reward feedback (ρ = −0.65, p_BH_ < 0.05, Fig. 3b), but not for anticipation (ρ = 0.3, p_BH_ = 0.26, Fig. 3a). On the other hand, 5-HT synthesis of the anterior insula was negatively correlated with reward anticipation (ρ = −0.61, p_BH_ < 0.05, Fig. 3c), but not with feedback (ρ = −0.45, p_BH_ = 0.16, Fig. 3d).

Finally, fPET and fMRI signal changes were used to explain behavioral differences in the reward task. Multivariable regression revealed that 5-HT synthesis of the anterior insula (beta = −23.6 s/min, ρ = −0.60, p_BH_ < 0.05), but not BOLD signal changes (p_BH_ > 0.15), was associated with the individual overall reaction time. No such relationship was observed for the VStr (all p > 0.4).

## Discussion

This work adapts fPET imaging and the synthesis model [16] to detect reward-specific changes in human 5-HT synthesis rates. Monetary gain and loss both elicited robust increases in 5-HT synthesis in both the VStr and anterior insula of 20–40% from baseline, respectively. Simultaneously acquired BOLD fMRI further revealed that 5-HT activity in the anterior insula was negatively associated with reward anticipation, whereas VStr 5-HT synthesis was negatively related to the processing of reward feedback. These findings provide novel insights into the regionally specific involvement of the serotonergic system in different phases of reward processing, thereby providing a promising approach to investigate pathological alterations in various patient cohorts.

Previous work has highlighted the importance of measuring 5-HT signaling in psychiatric disorders [28], with intensive research to discover non-invasive indices reflecting transient changes in the 5-HT system during cognitive or emotional stimulation [9, 10]. Considering the recent extensive debate on the involvement of 5-HT in MDD [29–35], it appears crucial to further clarify the underlying neurophysiological mechanisms. With respect to the current study, MDD patients (and medication-free patients in particular) exhibit reduced tryptophan levels [36] and decreased 5-HT synthesis as measured with [^11^C]AMT [37]. Furthermore, the recent application of a 5-HT_2A_ receptor agonist radioligand provided an essential cornerstone observation for decreased 5-HT release in MDD patients [38]. However, the mentioned study included a potent pharmacological challenge and characterized release capacity only at resting state. In contrast, our fPET approach assessing task-specific changes in 5-HT synthesis, uniquely enables the opportunity to track neurotransmitter action during the emotional process that is impaired in MDD patients (in this case reward). As a result, robust task-induced increases in 5-HT synthesis across different brain regions involved in reward processing were observed. Interestingly, reward-specific changes in 5-HT synthesis in the anterior insula were about twice as high compared to the VStr (40 vs 20%, respectively). The regional specificity of task-induced changes is supported by previous work demonstrating protein synthesis at nerve terminals [39, 40] and also highlights the importance of brain regions beyond the VStr in reward processing [5, 7].

Anatomically, the nucleus accumbens receives strong input from the insular cortex [41, 42]. Functionally, it has been shown that anticipatory activation in the insula encodes avoidance behavior in financial stimulation paradigms [43]. This aligns with suggestions that cortical activations are relevant for translating hedonic signals into cognitive counterparts [6, 44] and with impaired recruitment of cortical areas in anhedonia [45, 46]. For instance, MDD patients exhibit decreased activation of the VStr, but increased response in the orbitofrontal cortex [2], indicating a dysregulated interaction in the corticostriatal reward circuit. Similarly, connections of the insula to the VStr play an important role in addiction and drug-induced rewards [47]. The insular cortex is involved in drug craving [48] and experimental inhibition of this region [49] and its projections to the VStr specifically decreased drug abuse behavior [50, 51]. Together, these findings emphasize the interactions of the VStr as hedonic hotspot with other brain areas. Our results further specify this by highlighting the regionally distinct involvement of the 5-HT system in the reward circuitry.

Compared to our previous observations on dopamine synthesis changes [16], serotonin synthesis did not differ between monetary gain and loss, nor was there a sex-specific interaction with these conditions. These differences are particularly relevant when considering the distinct roles of the two neurotransmitter systems in reward and punishment processing [52]. Previous views of an opponency, with dopamine and serotonin coding for reward and punishment, respectively [53], have been extended to more complex interactions and even cooperation between the two neurotransmitters [52, 54]. This has led to the view that an additional role of serotonin in reward processing may be that of inhibition. On the other hand, it has been demonstrated multiple times that 5-HT neurons also positively encode reward behavior [8, 55, 56]. These different effects have previously been summarized in the sense that 5-HT initially activates the reward circuit, while on the long run it is responsible for inhibitory action [54]. Accordingly, our data suggests that immediate 5-HT action is indeed inhibitory on a neuronal level (negative association of 5-HT synthesis and BOLD signal), but activates reward behavior (higher 5-HT synthesis related to faster reaction time). These seemingly opposing effects may also be related to the underlying serotonin receptor subtypes [57, 58]. For instance, the insula contains highest levels of the 5-HT_1A_ receptor, the major inhibitory subtype [59], while the VStr is rich in the excitatory 5-HT_4_ [60] and 5-HT_6_ receptors [61]. Accordingly, specific patterns of disturbed receptor expressions have been observed in psychiatric disorders, with multiple alterations in MDD and schizophrenia, but more selective ones in addiction [62].

Regarding the association between task-specific changes in 5-HT synthesis and the BOLD signal, it is important to emphasize that these represent complementary aspects of neuronal activation. Unlike other radioligands that cross the BBB through passive diffusion, tryptophan and [^11^C]AMT are transported via a facilitated mechanism mediated by the L-type amino acid transporter 1 (LAT1) [63, 64]. This mechanism is similar to that of other radioligands such as 6-[^18^F]FDOPA and [^18^F]FDG with the latter being transported by glucose transporter 1. These transport molecules tightly regulate BBB passage and maintain a concentration gradient [64]. Crucially, this regulated transport results in a low extraction rate of the radiotracer from blood plasma, approximately 2% for [^11^C]AMT [22]. Consequently, typical increases in cerebral blood flow (approximately 25% [65–67]), which are key drivers of the BOLD signal, will produce only a 0.5% change in the [^11^C]AMT PET signal. Furthermore, if [^11^C]AMT merely reflected the BOLD fMRI signal, correlations between these imaging metrics would be ubiquitous. However, our findings indicate that this is not the case, as correlations were specific to certain brain regions and task conditions (Fig. 3).

As a limitation, we are aware that the radioligand [^11^C]AMT may enter the kynurenine pathway. So far, this has been mostly relevant for the investigation of pathological conditions such as brain tumors or epileptogenic regions [68]. Thus, it is not expected to affect 5-HT synthesis rates in healthy subjects [69], particularly when considering that the concentrations of 5-HT and its main metabolite is about 20-200 times higher than the metabolites of the kynurenine pathway [70, 71]. In line, activation of the kynurenine pathway by interferon-α did not affect cerebral spinal fluid tryptophan concentrations [72]. Still, we acknowledge impairments of the kynurenine pathway in psychiatric disorders [73] and that the kynurenine to tryptophan ratio may be shifted in sub-groups of MDD and schizophrenia [74]. However, these alterations are present at baseline, whereas our approach specifically targets task-induced changes in tryptophan metabolism. Thus, even in patient cohorts dynamic stimulation effects are unlikely to be affected by non-acute inflammatory processes when considering the short time scale of a PET scan of 60 min. As an advantage, [^11^C]AMT binds at the rate-limiting enzyme tryptophan hydroxylase and is not subject to metabolism by monoamine oxidase A, in contrast to [^11^C]5-HTP [75]. A further limitation is the relatively small sample size of this study. Thus, it is possible that more subtle changes in other reward-specific brain regions were not discovered in the voxel-wise analysis. Nevertheless, the effects observed in the VStr and anterior insula were sufficiently pronounced to elicit statistically robust increases in 5-HT synthesis. Although the current study only used reward stimuli, it is reasonable to assume that the approach can be extended to other cognitive and emotional domains, given the successful application of fPET for various different paradigms [23, 25, 76].

In conclusion, we established a feasible and robust approach to identify the dynamics of 5-HT synthesis during emotional stimulation in the human brain. This offers a promising approach to track pathological alterations in various brain disorders during the execution of exactly those emotional processes that are impaired, yielding a specifically tailored marker of altered 5-HT signaling.

## Data Availability

Raw data will not be publicly available due to reasons of data protection. Processed data and custom code can be obtained from the corresponding author with a data-sharing agreement, approved by the departments of legal affairs and data clearing of the Medical University of Vienna.
